# Dual Light Emission of CsSnI_3_-Based Powders Synthesized via a Mechanochemical Process

**DOI:** 10.3390/ma17143577

**Published:** 2024-07-19

**Authors:** Xuan Huang, Xiaobing Tang, Xiyu Wen, Yuebin Charles Lu, Fuqian Yang

**Affiliations:** 1 Laboratory of Functional Materials, University of Kentucky, Lexington, KY 40506, USA; xuan.huang@uky.edu (X.H.); xbtang@usst.edu.cn (X.T.); 2 Department of Mechanical and Aerospace Engineering, University of Kentucky, Lexington, KY 40506, USA; 3 Department of Chemical and Materials Engineering, University of Kentucky, Lexington, KY 40506, USA; 4 School of Mechanical Engineering, University of Shanghai for Science and Technology, Shanghai 200093, China; 5 Center for Aluminium Technology, University of Kentucky, Lexington, KY 40506, USA; xwen2@uky.edu

**Keywords:** mechanochemical synthesis, lead-free perovskites, CsSnI_3_, dual light emission

## Abstract

Lead toxicity has hindered the wide applications of lead halide perovskites in optoelectronics and bioimaging. A significant amount of effort has been made to synthesize lead-free halide perovskites as alternatives to lead halide perovskites. In this work, we demonstrate the feasibility of synthesizing CsSnI_3_-based powders mechanochemically with dual light emissions under ambient conditions from CsI and SnI_2_ powders. The formed CsSnI_3_-based powders are divided into CsSnI_3_-dominated powders and CsSnI_3_-contained powders. Under the excitation of ultraviolet light of 365 nm in wavelength, the CsSnI_3_-dominated powders emit green light with a wavelength centered at 540 nm, and the CsSnI_3_-contained powders emit orange light with a wavelength centered at 608 nm. Both the CsSnI_3_-dominated and CsSnI_3_-contained powders exhibit infrared emission with the peak emission wavelengths centered at 916 nm and 925 nm, respectively, under a laser of 785 nm in wavelength. From the absorbance spectra, we obtain bandgaps of 2.32 eV and 2.08 eV for the CsSnI_3_-dominated and CsSnI_3_-contained powders, respectively. The CsSnI_3_-contained powders exhibit the characteristics of thermal quenching and photoelectrical response under white light.

## 1. Introduction

The success in the synthesis of lead halide perovskites at low cost has opened active research fields for the applications of lead halide perovskites in photovoltaics and sensing and light-emitting devices and systems [1,2]. One of the challenges for the applications of lead halide perovskites is the lead toxicity, which has hindered the commercialization of lead halide perovskites. This has stimulated extensive work to explore the replacement of lead halide perovskites with lead-free halide perovskites and lead-free halide double perovskites. Sn-based halide perovskites represent one important group of lead-free halide perovskites.

There are various methods available to synthesize Sn-based halide perovskites, including spin-coating [3], vapor-assisted deposition [4], solid-phase sintering [5], hot injection [6], and mechanochemical processing [7,8]. Weiβ et al. [9] used atomic layer deposition and pulsed chemical vapor deposition to form γ-CsSnI_3_ film on patterned silicon substrates and obtained a bandgap of 1.20 eV from the Tauc plot. Shum et al. [10] used a two-step method to obtain CsSnI_3_ films on three different substrates of glass, ceramics, and silicon with thermal and electron-beam evaporators in a vacuum chamber (~10^5^ Torr). Kim and Kang [11] combined spin-cast with drop cast to form CsSnI_3_ crystals in a glove box with nitrogen. Wang et al. [12] used a one-pot process to synthesize CsSnI_3_ nanocrystals with and without the use of an antioxidant solvent additive (TPPi) and the prepared CsSnI_3_ nanocrystals to form CsSnI_3_ films. Kapil et al. [3] used a spray-deposition method to prepare Cs_2_SnI_6_ films and obtained a bandgap of 1.54 eV. Murshed and Bansal [13] applied drop-coating to construct Cs(Sn,Pb)I_3_ films with a bandgap of 1.5 eV for the applications in perovskite-based solar cells. Nairui et al. [14] synthesized Cs_2_SnI_6_ powders via a one-step method and studied dependence of the optical characteristics of the Cs_2_SnI_6_ powders on the process parameters, including reactant type and solvent type. Jiang et al. [15] used a chemical bath method to prepare Cs_2_SnI_6_ powders and reported the long-term stability of the Cs_2_SnI_6_ powders over a period of one month. Lee et al. [16] used electrospraying to form Cs_2_SnI_6−x_Br_x_ layers and evaluated the Br effect on the bandgaps of the Cs_2_SnI_6-x_Br_x_ layers. Saparov et al. [17] prepared Cs_2_SnI_6_ films via a two-step process and obtained a bandgap of 1.60 eV. Tang et al. [18] obtained Cs_2_SnI_6_ with a size-dependent light emission via an aqueous process. It is worth noting that extensive work has been conducted on the applications of Cs-, Sn-, and Sn-Pb-based perovskites in the field of solar cells. There are few works on infrared emission and green emission of Sn-based halide perovskites. Also, most processes reported in the literature to prepare Sn-based halide perovskites are much more complex than the mechanochemical process used in this work.

Currently, few studies [8,19] are on the use of mechanochemical processing to synthesize Sn-based halide perovskites. To realize the potential applications of Sn-based halide perovskites in solar cells and bioimaging, we used a mechanochemical method to synthesize CsSnI_3_-based powders under ambient conditions from CsI and SnI_2_ powders. In contrast to the work reported in the literature, the temporal evolution of the optical characteristics of the prepared CsSnI_3_-based powders were characterized. The prepared CsSnI_3_-based powders were divided into two groups—one is CsSnI_3_-dominated powders, which emit green light under the excitation of UV light of 365 nm, and the other is CsSnI_3_-contained powders, which emit orange light under the excitation of UV light of 365 nm. Both the CsSnI_3_-dominated powders and CsSnI_3_-contained powders exhibit infrared emission with the emission wavelengths centered at 916 nm and 925 nm, respectively, under a laser of 785 nm in wavelength.

## 2. Experimental Details

The materials used to prepare Sn-based halide perovskites are cesium iodide (CsI, 99.9%, Alfa Aesar, Ward Hill, MA, USA)**,** tin (Ⅱ) iodide (SnI_2_, 99%, Strem Chemical, Newburyport, MA, USA), and deionized (DI) water. The as-received materials were used directly in the experiments without pre-purification.

**Figure 1:**
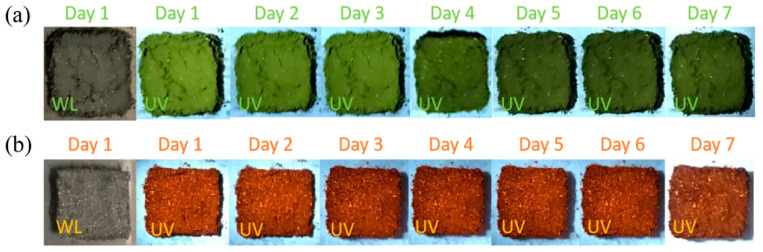
Optical images of the prepared powders under white light and UV light of 365 nm in wavelength: (**a**) CsSnI_3_-dominated powders, and (**b**) CsSnI_3_-contained powders.

For the synthesis of CsSnI_3_-dominated powders, 0.4 mmol (103.92 mg) CsI was mixed with 0.4 mmol (149 mg) SnI_2_ to form a mixture without any additives and water. The mixture was put in a ceramic mortar and ground mechanically at ~18.4 °C and with a humidity of ~22% for 5 min initially. Twenty milliliters of DI water was then added to the ground material, which was further ground mechanically for 5 min, leading to the formation of black powders (CsSnI_3_-dominated powders) under white light, as shown in Figure 1a. The as-prepared black powders were stored in a vacuum chamber (Napco 5831) at 44.7 KPa for one week.

For the synthesis of CsSnI_3_-contained powders, 0.2 mmol (51.96 mg) CsI was mixed with 0.2 mmol (74.5 mg) SnI_2_ with 200 μL of DI water. The mixture was put in a ceramic mortar and ground mechanically at ~18.4 °C and with a humidity of ~22% until the color of the mixture became light black. The ground mixture was dried naturally to form light black powders (CsSnI_3_-contained powders) in the mortar under white light, as shown in Figure 1b.

The crystal structures of the prepared CsSnI_3_-based powders were analyzed on an X-ray analyzer (Bruker D8 Discover, Bruker, Billerica, MA, USA) with a scintillation counter detector under the CuKα radiation of λ = 1.5406 Å. The chemical compositions and morphologies of the prepared CsSnI_3_-based powders were characterized on a scanning electron microscope (SEM) (JEOL JSM-5900lLV, JEOL, Tokyo, Japan) equipped with an EDS microanalysis system.

A spectrometer (Ocean Optics, FLAME-S-VIS-NIR-ES, Ocean Optics, Orlando, FL, USA) was used to characterize the photoluminescence (PL) of the synthesized CsSnI_3_-based powders under the excitation of UV light of 365 nm in wavelength and a laser of 785 nm in wavelength, respectively. The absorbance of the synthesized CsSnI_3_-based powders was analyzed on a UV-Visible spectrophotometer (EVOLUTION 201, Thermo Fisher scientific, Waltham, MA, USA). The photoconduction of the prepared CsSnI_3_-based powders was examined on a power meter (Keithley 2400, Keithley, Solon, OH, USA) under white light.

## 3. Results and Discussion

Figure 1 depicts optical images of the synthesized CsSnI_3_-dominated powders, which were stored in a vacuum chamber, and the prepared CsSnI_3_-contained powders, which were placed under ambient conditions, under UV light of 365 nm in wavelength over a period of 7 days. It is evident that the color of the CsSnI_3_-based powders under the UV light experienced gradual changes from light green to dark green for the CsSnI_3_-dominated powders and from light orange to dark orange for the CsSnI_3_-contained powders over the period of 7 days. The green emission of CsSnI_3_-based powders under UV light of 365 nm in wavelength has not been reported in the literature to our knowledge. Note that Bharti et al. [20] recently reported the formation of green CsSnI_3_ via solid state reaction under mechanical grounding and sintering at 280 K. The orange emission of the CsSnI_3_-contained powders is qualitatively in accord with the light emission of Cs_2_SnI_6_ nanobelts/nanocrystals in mother liquor reported by Wang et al. [21]. Note that the Cs_2_SnI_6_ nanobelts/nanocrystals reported in the work of Wang et al. [21] were synthesized by a hot injection method with the use of octadecene, oleylamine, and oleic acid, which is significantly different from the method used in this work.

**Figure 2:**
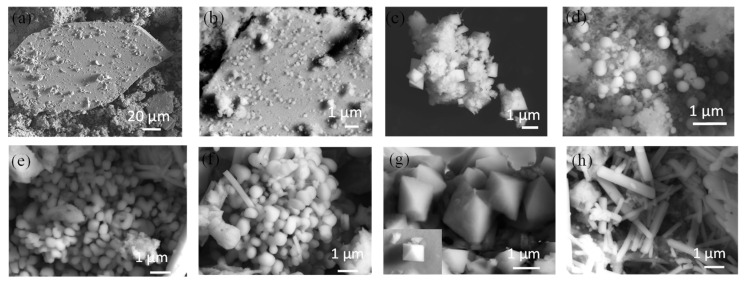
SEM images of green-emitting powders: (**a**,**b**) plate-like structure, (**c**) Cs_2_SnI_6_ octahedral microcrystals, and (**d**) spherical nanocrystals; SEM images of orange-emitting powders: (**e**,**f**) spherical nanocrystals, (**g**) octahedral microcrystals, and (**h**) rod-like microcrystal.

Figure 2a–d depicts SEM images of green-emitting powders. There are plate-like structures formed from the grounding of the mixture, as shown in Figure 2a,b, which can be attributed to the combinational effect of the shear and compression deformation. There are small particles on the surfaces of the plate-like structures. The SEM images in Figure 2c,d show the formation of aggregates of particles in a nearly octahedral shape and in a nearly spherical shape, respectively. The size of the particles in a nearly octahedral shape is in a range of 100 nm to 300 nm, and the size of the particles in a nearly spherical shape is in a range of 380 nm to 880 nm. The EDS analyses, as shown in Figures S1–S3 in Supplementary Information, yield atomic ratios of nearly 1:1:3 of Cs:Sn:I, 1:1:3 of Cs:Sn:I, 2:1:6 of Cs:Sn:I, and 1:1 of Cs:I for the plate-like structures, the particles on the surface of the plate-like structures, the particles in a nearly octahedral shape, and the particles in a nearly spherical shape, respectively. This result indicates that the prepared green-emitting powders consist of CsSnI_3_, Cs_2_SnI_6_, and CsI crystals. Note that the octahedral shape of Cs_2_SnI_6_ crystal is consistent with the observation reported in the literature [18,22,23].

Figure 2e–h depicts SEM images of the orange-emitting powders. There are many particles presented in a bean-like or sphere-like shape from the grounding of the mixture, as shown in Figure 2e,f, in contrast to the plate-like structures for the green-emitting powders. The SEM images in Figure 2g,h show particles in an octahedral shape and in a rod shape, respectively. The size of the particles in a bean-like or sphere-like shape is in a range of 250 nm to 880 nm, and the size of the particles in an octahedral shape is in a range of 500 nm to 1 μm. The dimensions of the rod-like particles are 500 nm to 4 μm in length and 157 nm to 587 nm in diameter. The EDS analyses, as shown in Figures S4–S6 in Supplementary Information, yield atomic ratios of nearly 1:1 of Cs:I, 2:1:6 of Cs:Sn:I, and 1:1:3 of Cs:Sn:I for the particles in a bean-like or sphere-like shape, the particles in an octahedral shape, and the rod-like particles, respectively. This result indicates that the prepared orange-emitting powders consist of CsSnI_3_, Cs_2_SnI_6_, and CsI crystals.

**Figure 3:**
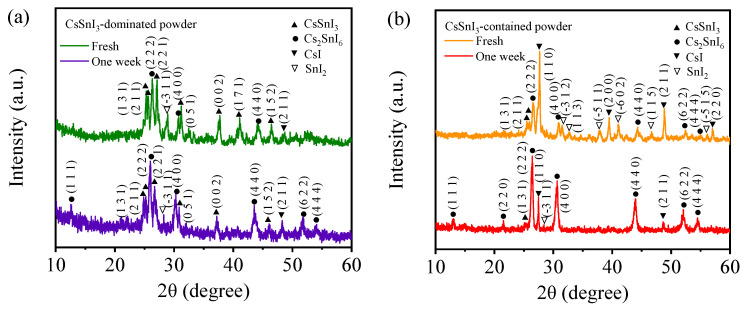
XRD patterns of (**a**) freshly prepared CsSnI_3_-dominated powders and the one stored in a vacuum chamber for one week, and (**b**) freshly prepared CsSnI_3_-contained powders and the one stored under ambient conditions for one week.

Figure 3 presents the XRD patterns of the freshly prepared CsSnI_3_-dominated powders and CsSnI_3_-contained powders and the corresponding ones stored for one week. According to Figure 3a, there are diffraction peaks corresponding to CsSnI_3_, Cs_2_SnI_6_, SnI_2_, and CsI for the freshly prepared CsSnI_3_-dominated powders, which are in accord with the EDS results presented in the supplementary information. Specifically, the diffraction peaks centered at 25.52°, 25.90°, 31.48°, 37.73°, 41.36°, and 46.78° correspond to the crystal planes of (131), (211), (221), (051), (002), (171), and (152) of orthorhombic CsSnI_3_ (PDF#97-001-4070); the diffraction peaks centered at 26.53°, 30.73°, and 44.02° correspond to the crystal planes of (222), (400), and (440) of cubic Cs_2_SnI_6_ (PDF#97-025-0743); the peak centered at 48.80° corresponds to the crystal plane of (211) of cubic CsI (PDF#97-004-4938); and the diffraction peak centered at 28.67° corresponds to the crystal plane of ($\overset{-}{3}$11) of monoclinic SnI_2_ (PDF#97-000-2831). Using the XRD peaks, the molar fractions of individual compounds in the freshly prepared CsSnI_3_-dominated powers are calculated and listed in Table 1. It is evident that CsSnI_3_ has the largest molar fraction of 32.80%. Thus, the green-emitting powders are referred to as CsSnI_3_-dominated powders. The larger molar fraction of SnI_2_ than CsI in the freshly prepared powders indicates that more CsI reacted to form CsSnI_3_ and Cs_2_SnI_6_ than SnI_2_ during the grinding. It is speculated that the green emission of the powders may be associated with the doping in SnI_2_.

The XRD pattern of the CsSnI_3_-dominated powders stored in a vacuum chamber for one week is also depicted in Figure 3a. The diffraction peaks centered at 25.43°, 25.80°, 27.317.31°, 31.48°, 37.82°, and 46.70° correspond to the crystal planes of (131), (211), (221), (051), (002), and (152) of orthorhombic CsSnI_3_ (PDF#97-001-4070); the diffraction peaks centered at 13.15°, 26.58°, 30.89°, 44.13°, 52.38°, and 54.62° correspond to the crystal planes of (111), (222), (400), (440), (622), and (444) of cubic Cs_2_SnI_6_ (PDF#97-025-0743); the peak centered at 48.84° corresponds to the crystal plane of (211) of cubic CsI (PDF#97-004-4938); and the diffraction peak centered at 28.87° corresponds to the crystal plane of ($\overset{-}{3}$11) of monoclinic SnI_2_ (PDF#97-000-2831). There are no diffraction peaks of new phases presented in the figure in comparison with the freshly prepared one. Using the XRD peaks, the molar fractions of individual compounds in the CsSnI_3_-dominated powders stored in the vacuum chamber for one week are calculated and listed in Table 1. It is evident that there are slight decreases in the molar fractions of both CsSnI_3_ and SnI_2_ and increases in the molar fractions of both Cs_2_SnI_6_ and CsI. The increase of Cs_2_SnI_6_ is likely from CsSnI_3_ and SnI_2_ with the following reactions:

2CsSnI_3_ + O_2_ → Cs_2_SnI_6_ + SnO_2_ (1)

2CsI + 2SnI_2_ + O_2_ → Cs_2_SnI_6_ + SnO_2_ (2)

There is a decrease in the molar fraction of SnI_2_ over the period of 7 days, suggesting that the doping effect becomes weaker, as indicated by the optical images in Figure 1a. Note that the presence of oxygen in the vacuum chamber was due to the limit of the vacuum pressure.

Following the method used by Misra et al. [24], the pseudo-cubic lattice constants of the freshly prepared and the stored CsSnI_3_-dominated powders are calculated from the XRD patterns in Figure 3a and listed in Table S1 in Supplementary Information. It is evident that the lattice constants of CsSnI_3_ and Cs_2_SnI_6_ increase slightly after being stored for one week.

The XRD pattern of the freshly prepared CsSnI_3_-contained powders is depicted in Figure 3b. The peaks centered at 26.54°, 30.90°, 44.31°, 52.44°, and 54.99° correspond to the crystal planes of (222), (400), (440), (622), and (444) of cubic Cs_2_SnI_6_ (PDF#97-025-0743); the peaks centered at 25.49° and 25.96° correspond to the crystal planes of (131) and (211) of orthorhombic CsSnI_3_ (PDF#97-001-4070); the peaks centered at 39.48°, 48.85°, and 57.02° correspond to the crystal planes of (200), (211), and (220) of cubic CsI (PDF#97-004-4938); and the peaks centered at 31.50°, 32.75°, 37.88°, 41.04°, 46.68°, and 56.18° correspond to crystal planes of ($\overset{-}{3}$12), (113), ($\overset{-}{5}$11), ($\overset{-}{6}$02), (115), and ($\overset{-}{5}$15) of monoclinic SnI_2_ (PDF#97-000-2831). This result indicates that the freshly prepared CsSnI_3_-contained powders consist of four phases of Cs_2_SnI_6_, CsSnI_3_, CsI, and SnI_2_. Using the XRD peaks, the molar fractions of individual compounds in the freshly prepared CsSnI_3_-contained powders are calculated and listed in Table 2. It is evident that CsI has the largest molar fraction of 73.63%. Thus, the orange-emission powders are referred to as CsSnI_3_-contained powders.

Figure 3b also shows the XRD pattern of the CsSnI_3_-contained powders stored under ambient conditions for one week. The peaks centered at 13.08°, 21.54°, 26.41°, 30.69°, 43.91°, 52.10°, and 54.49° correspond to the crystal planes of (111), (220), (222), (400), (440), (622), and (444) of cubic Cs_2_SnI_6_ (PDF#97-025-0743); the peaks centered at 27.44° and 48.64° correspond to the crystal planes of (110) and (211) of cubic CsI (PDF#97-004-4938); the peak centered at 25.52° corresponds to the crystal plane of (131) of orthorhombic CsSnI_3_ (PDF#97-001-4070); and the peak centered at 28.39° corresponds to the crystal plane of ($\overset{-}{3}$11) of monoclinic SnI_2_ (PDF#97-000-2831). There are no diffraction peaks of new phases presented in the figure in comparison with the freshly prepared one. Using the XRD peaks, the molar fractions of individual compounds in the CsSnI_3_-contained powders stored under ambient conditions for one week are calculated and listed in Table 2. It is evident that there are slight decreases in the molar fractions of both CsSnI_3_ and SnI_2_, a large decrease in the molar fraction of CsI, and a large increase in the molar fraction of Cs_2_SnI_6_. There are reactions that lead to the increase of Cs_2_SnI_6_ and the decrease in CsI in the CsSnI_3_-contained powders.

From Table 2, we note a large molar fraction of CsI in the CsSnI_3_-contained powders. The orange-emission of the CsSnI_3_-contained powders is likely associated with the doping of Sn in CsI, as illustrated in Figures S7–S9 in Supplementary Information.

The larger molar fraction of CsI compared to SnI_2_ in the CsSnI_3_-contained powders indicates that more SnI_2_ reacted to form CsSnI_3_ and Cs_2_SnI_6_ than CsI. The orange emission of the powders can be attributed to the doping in CsI. After one week, the molar fraction of Cs_2_SnI_6_ increases, while the molar fractions of SnI_2_, CsSnI_3_, and CsI decrease, suggesting that the Cs_2_SnI_6_ comes from CsSnI_3_ and SnI_2_ through the reactions illustrated in Equations (1) and (2). The decrease in the molar fraction of CsI means the doping effect is weaker as indicated by the optical images in Figure 1b.

**Figure 4:**
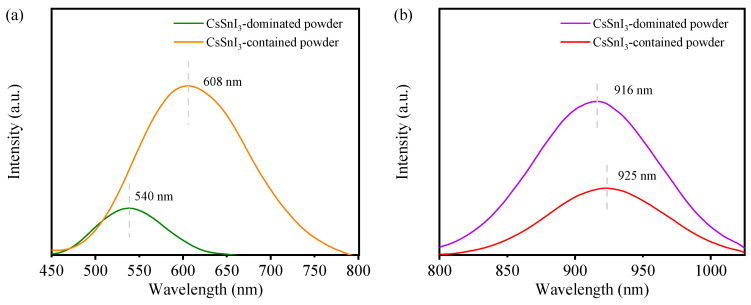
PL spectra of (**a**) freshly prepared CsSnI_3_-dominated and CsSnI_3_-contained powders under UV light of 365 nm in wavelength, and (**b**) freshly prepared CsSnI_3_-dominated and CsSnI_3_-contained powders under a laser of 785 nm in wavelength.

Figure 4a,b presents PL spectra of the freshly prepared CsSnI_3_-based powders under UV light of 365 nm and a laser of 785 nm, respectively. It is interesting to observe that the CsSnI_3_-dominated powders emit both green light centered at 540 nm in wavelength under the UV light and infrared light centered at 916 nm in wavelength under the 785 nm laser. The emission of the green light centered at 540 nm has not been reported in the literature, and the emission of the infrared light centered at 916 nm corresponds to a bandgap of 1.35 eV in accord with 1.31 eV reported by Chung et al. [25].

According to Figure 4a,b, the CsSnI_3_-contained powders emit orange light centered at 608 nm in wavelength under the UV light and infrared light centered at 925 nm in wavelength under the laser. The emission of the orange light centered at 608 nm is close to the PL emission of Cs_2_SnI_6_ centered at 620 nm reported by Wang et al. [21], who prepared Cs_2_SnI_6_ nanocrystals with a hot-injection process. The emission of the infrared light centered at 925 nm corresponds to a bandgap of 1.34 eV in accord with 1.32 eV reported by Wang et al. [26]. The PL peak intensity for the green emission is significantly lower than that for the orange emission, and the PL peak intensity for the infrared emission centered at 916 nm is higher than that for the infrared emission centered at 925 nm. Such differences suggest that the CsSnI_3_-dominated powders (green emission) and CsSnI_3_-contained powders (orange emission) possess different characteristics in terms of photoluminescence in the visible and infrared regimes, which can be attributed to the differences in compositions.

**Figure 5:**
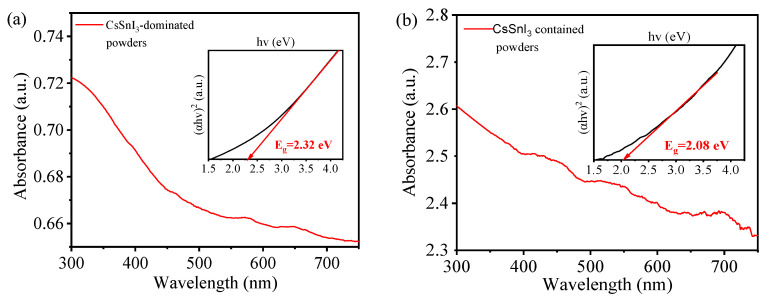
Absorbance spectra and Tauc plots (insets) of freshly prepared (**a**) CsSnI_3_-dominated powders and (**b**) CsSnI_3_-contained powders.

Figure 5a depicts the absorbance spectrum of the CsSnI_3_-dominated powders. There is no distinct band-edge absorption peak, which might be due to the contributions from four different compounds and the wide range of crystal sizes. Using the absorbance spectrum, the corresponding Tauc plot is constructed and shown as an inset in Figure 5a. From the Tauc plot, we obtain a bandgap of 2.32 eV, which is slightly larger than 2.29 eV from the PL spectrum of the CsSnI_3_-dominated powders under UV light of 365 nm and less than 2.55 eV reported by Chung et al. [25],

Figure 5b depicts the absorbance spectrum of the CsSnI_3_-contained powders. There is no distinct band-edge absorption peak, which is similar to the CsSnI_3_-dominated powders. Such behavior again might be due to the contributions from four different compounds and the wide range of crystal sizes. Using the absorbance spectrum, the corresponding Tauc plot is constructed and shown as an inset in Figure 5b. From the Tauc plot, we obtain a bandgap of 2.08 eV, which is slightly larger than 2.04 eV from the PL spectrum of the CsSnI_3_-contained powders under the excitation of UV light of 365 nm.

**Figure 6:**
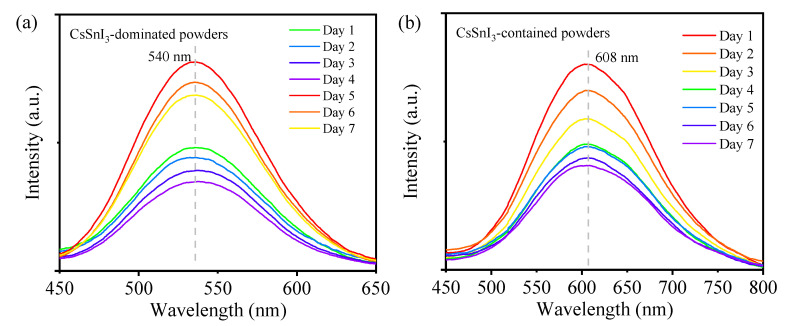
Long-term stability of the CsSnI_3_-based powders over a period of 7 days: (**a**) CsSnI_3_-dominated powders in a vacuum chamber, and (**b**) CsSnI_3_-contained powder under ambient conditions.

The long-term stability of the CsSnI_3_-based powders was examined over a period of 7 days. Figure 6a,b depicts the PL spectra of the CsSnI_3_-dominated powders and CsSnI_3_-contained powders, respectively. The emission wavelengths of both the powders remain unchanged. The peak intensity decreases continuously, which is consistent with the color change, as shown in Figure 1. Such a trend implies that the prepared CsSnI_3_-based powders are not at a “stable” state. There exist chemical reactions occurring in the CsSnI_3_-based powders over the period of 7 days, as supported by the changes in the molar fractions shown in Table 1 and Table 2. The chemical reactions lead to the evolution of individual compounds and result in the decrease in the PL peak intensity. Note that the oxidation of Sn occurred in the vacuum chamber and under ambient conditions, which caused the degradation of the CsSnI_3_-based powders responsible for the decrease in the PL peak intensity. The decreasing trend in the PL peak intensity with respect to time is similar to the decreasing trend for MAPbI_3_ films reported by Mahon et al. [27]. However, there exist differences in the observed behavior. Mahon et al. [27] used a continuous-wave laser as the excitation source and focused their study on a short time period of 250 s. It is also noted that continuous illumination of a laser can cause the variation of the concentration of charger carriers and local temperature [28], leading to the change of the PL peak intensity.

**Figure 7:**
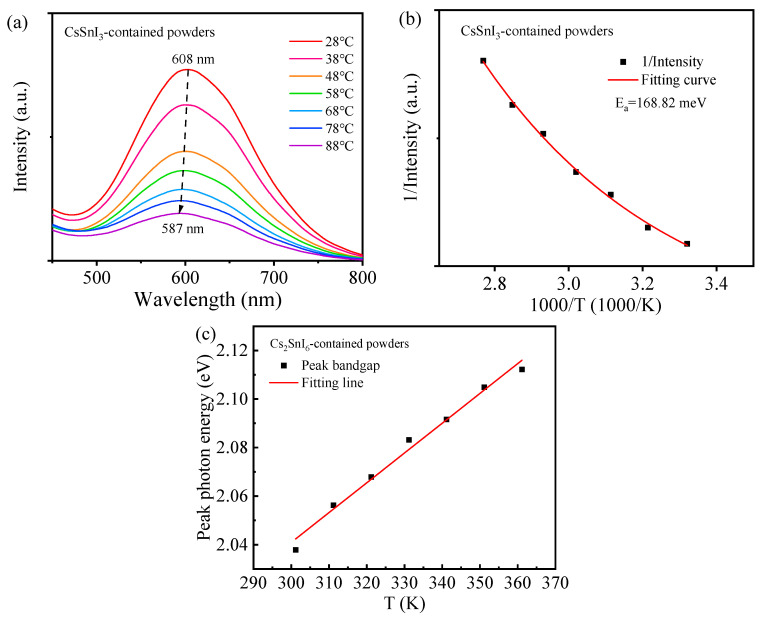
Temperature effects on the PL characteristics of the CsSnI_3_-contained powders: (**a**) PL spectra at different temperatures, (**b**) PL intensity vs. temperature, and (**c**) photon energy vs. temperature.

Figure 7a depicts the PL spectra of the CsSnI_3_-contained powders at different temperatures, from which we determine the peak wavelength and peak intensity at different temperatures. The PL peak wavelength experiences a blue shift from 608 nm to 587 nm when the temperature was increased from 28 °C to 88 °C, and increasing the temperature causes the decrease of the peak intensity. Figure 7b,c shows the temperature dependence of the peak intensity and peak wavelength, respectively.

In general, the variation of the peak intensity of the emission light with temperature can be expressed as [29]

(3) $$I(T)=\frac{I_{0}}{{\mathrm{Ae}}^{\left(-E_{a}/k_{B}T\right)}+1}$$

where *I*_0_ is the peak intensity at 0 K, *E_a_* is the activation energy, *k_B_T* is the thermal energy, and A is a constant. Using Equation (3) to fit the data in Figure 7b yields the activation energy of 168.82 meV. For comparison, we include the fitting curve in Figure 7b. Such a large value of the activation energy suggests that the decrease in the PL peak intensity is ascribed to thermal quenching.

According to Yang [30] and Tang et al. [31], the relation between temperature, *T*, and the bandgap of a semiconductor, *E*, for |Δ*T*| << *T*_0_ can be formulated to the first-order approximation as

(4) $$E(T)=E(T_{0})+\alpha (T-T_{0})$$

with *T*_0_ as a reference temperature, and α as a constant representing the temperature dependence of the bandgap. According to Figure 7b, the bandgap increases linearly with increasing temperature, supporting Equation (3) with α > 0. The positive value of α implies that there exists electron–phonon interaction, which widens the bandgap of the CsSnI_3_-contained powders [31].

It must be pointed out that the CsSnI_3_-dominated powders are unstable after being exposed to the air for a short period. Thus, no experiments were conducted on the thermal stability of the CsSnI_3_-dominated powders.

**Figure 8:**
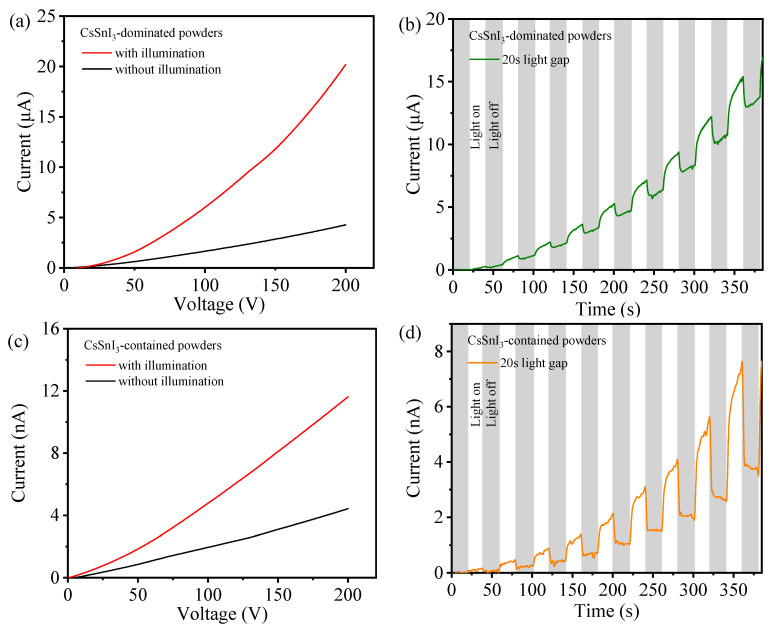
Photo-response of the CsSnI_3_-dominated powders during a voltage sweeping from 0 V to 200 V (**a**) with and without the illumination of white light and (**b**) under a 20 s light on-and-off cycle for 385 s; photo-response of the CsSnI_3_-contained powders during a voltage sweeping from 0 V to 200 V, (**c**) with and without the illumination of white light and (**d**) under a 20 s light on-and-off cycle for 385 s.

The photo-responses of the CsSnI_3_-dominated powders and CsSnI_3_-contained powders were investigated using a Keithley 2400 sourcemeter. A total of 0.4 mmol of the prepared powders was placed onto a glass substrate. The powders were then shaped to a 1 × 1 cm^2^ shape and connected to a pair of copper electrodes. The photo-response of the powders was evaluated under a voltage sweeping at an increment of 84 mV. Figure 8a presents the I–V curve of the CsSnI_3_-dominated powder with and without the illumination of white light. Increasing the voltage leads to a nonlinear increase of the current. The illumination of white light significantly increases the current through the powders, revealing the presence of optoelectrical response under the illumination of white light. Figure 8b presents the I-V curve of the CsSnI_3_-dominated powders under the periodic on-and-off of white light at a time interval of 20 s. It is evident that the illumination of white light reduced the resistance to the current consistent with the results in Figure 8a. The CsSnI_3_-dominated powders exhibit lucrative photoconductive characteristics, suggesting the potential application in perovskite-based solar cells.

Figure 8c,d presents the I-V curve of the CsSnI_3_-contained powder with and without the illumination of white light and the I-V curve of the CsSnI_3_-contained powders under the periodic on-and-off of white light at a time interval of 20 s, respectively. Similarly, the CsSnI_3_-contained powders also exhibit photoconductive characteristics. Comparing Figure 8c,d to Figure 8a,b, we can conclude that the CsSnI_3_-dominated powders have significantly higher photoconductivity than the CsSnI_3_-contained powders, indicating a higher photon-generated carrier concentration in the CsSnI_3_-dominated powders than the CsSnI_3_-contained powders. Note that similar photoconductive behavior was also observed in MAPbI_3_ by Khenkin et al. [32], who suggested that the variation of dark conductivity is attributed to the phase change and photoconductivity has a non-monotonic dependence on temperature.

## 4. Conclusions

Producing lead-free halide perovskites is of practical importance for their applications in solar cells and bioimaging to avoid the detrimental effects of lead to the planet and human health. We have demonstrated that the mechanical grounding of CsI and SnI_2_ under two different conditions can produce CsSnI_3_-based powders, which can exhibit dual light emission under two different lights of 365 nm and 785 nm in wavelength. Such a process avoids the use of toxic, organic solvents and can be scaled up to produce Sn-based halide perovskites for industrial applications. The prepared CsSnI_3_-based powders were divided into two groups—one is the CsSnI_3_-dominated powders, and the other is the CsSnI_3_-contained powders. Both powders consist of four different chemical compounds of CsI, SnI_2_, CsSnI_3_, and Cs_2_SnI_6_. Under the excitation of UV light of 365 nm, the CsSnI_3_-dominated powders exhibit green emission with the wavelength centered at 540 nm, and the CsSnI_3_-contained powders exhibit orange emission with the wavelength centered at 608 nm. Such a large difference in the emission wavelength is likely due to the differences in the molar fractions of the four different chemical compounds. Under a laser of 785 nm, the CsSnI_3_-dominated powders exhibit infrared emission with the wavelength centered at 916 nm, and the CsSnI_3_-contained powders exhibit infrared emission with the wavelength centered at 925 nm. However, the lower emission intensity and stability of the prepared Sn-based halide perovskites than lead-based perovskites likely hinder their applications in solar cells.

The analysis of the thermal stability of the CsSnI_3_-contained powders yielded an activation energy of 168.82 meV. Thermal quenching plays a crucial role in determining the stability of the CsSnI_3_-contained powders at elevated temperatures. Both the CsSnI_3_-dominated powders and the CsSnI_3_-contained powders exhibit photoconductive characteristics with a decrease in the resistance of the powders under white light. The CsSnI_3_-dominated powders have significantly higher photoconductivity than the CsSnI_3_-contained powders.

## Figures and Tables

**Table 1:** Molar ratios of four compounds in the CsSnI_3_-dominated powders.

	CsI	SnI_2_	CsSnI_3_	Cs_2_SnI_6_
Fresh	20.93%	27.86%	32.80%	18.41%
Week 1	28.53%	24.04%	26.68%	20.75%

**Table 2:** Molar ratios of four compounds in the CsSnI_3_-contained powders.

	CsI	SnI_2_	CsSnI_3_	Cs_2_SnI_6_
Fresh	74.11%	10.59%	7.68%	7.62%
Week 1	56.82%	8.65%	6.38%	28.16%

## Data Availability

The original contributions presented in the study are included in the article/supplementary material, further inquiries can be directed to the corresponding authors.

## Supplementary Materials

The following supporting information can be downloaded at: https://www.mdpi.com/article/10.3390/ma17143577/s1, Figure S1: EDS spectrum of CsSnI_3_ plate. (In Spectrum 14, atomic ratio of Cs: Sn: I = 21.73%: 19.96%: 58.31%); Figure S2. EDS spectrum of Cs_2_SnI_6_ plate. (In Spectrum 21, atomic ratio of Cs: Sn: I = 24.1%: 12.6%: 63.3%); Figure S3. EDS spectrum of CsI crystals. (In Spectrum 15, atomic ratio of Cs: I = 49.80%: 45.90%); Figure S4. EDS spectrum of CsI crystals. (In Spectrum 9, atomic ratio of Cs: I = 50.40%: 44.90%); Figure S5. EDS spectrum of CsI crystals. (In Spectrum 1, atomic ratio of Cs:Sn: I = 23.34%: 14.46%: 62.20%); Figure S6. EDS spectrum of CsI crystals. (In Spectrum 11, atomic ratio of Cs: Sn: I = 21.7%: 20.9%: 57.4%); Figure S7. Optical image of SnI2-doped CsI under white light and UV lamp (365 nm); Figure S8. Photoluminescence of SnI2-doped CsI by mechanical synthesis excited under: (a) UV lamp (365 nm); (b) 785 nm laser at 0.75 V; Figure S9. XRD pattern of SnI2-doped CsI by mechanical synthesis. Table S1: Lattice constants of freshly prepared and stored CsSnI3-based powders.

## References

[1] Ricciardulli A.G., Yang S., Smet J.H., Saliba M. (2021). Emerging perovskite monolayers. Nat. Mater..

[2] Green M.A., Ho-Baillie A., Snaith H.J. (2014). The emergence of perovskite solar cells. Nat. Photonics.

[3] Kapil G., Ohta T., Koyanagi T., Vigneshwaran M., Zhang Y., Ogomi Y., Pandey S.S., Yoshino K., Shen Q., Toyoda T. (2017). Investigation of interfacial charge transfer in solution processed Cs_2_SnI_6_ thin films. J. Phys. Chem. C.

[4] Lee B., Shin B., Park B. (2019). Uniform Cs_2_SnI_6_ thin films for lead-free and stable perovskite optoelectronics via hybrid deposition approaches. Electron. Mater. Lett..

[5] Zhang J., Li S., Yang P., Liu W., Liao Y. (2018). Enhanced stability of lead-free perovskite heterojunction for photovoltaic applications. J. Mater. Sci..

[6] Dolzhnikov D.S., Wang C., Xu Y., Kanatzidis M.G., Weiss E.A. (2017). Ligand-free, quantum-confined Cs_2_SnI_6_ perovskite nanocrystals. Chem. Mater..

[7] Cho H., Yun Y., Choi W.C., Cho I.S., Lee S. (2022). Structural, optical, and electrical properties of tin iodide-based vacancy-ordered-double perovskites synthesized via mechanochemical reaction. Ceram. Int..

[8] El Ajjouri Y., Locardi F., Gélvez-Rueda M.C., Prato M., Sessolo M., Ferretti M., Grozema F.C., Palazon F., Bolink H.J. (2020). Mechanochemical synthesis of Sn (II) and Sn (IV) iodide perovskites and study of their structural, chemical, thermal, optical, and electrical properties. Energy Technol..

[9] Weiss A., Terletskaia M., Popov G., Mizohata K., Leskelä M., Ritala M., Kemell M. (2023). Atomic layer deposition and pulsed chemical vapor deposition of SnI_2_ and CsSnI_3_. Chem. Mater..

[10] Shum K., Chen Z., Qureshi J., Yu C.L., Wang J.J., Pfenninger W., Vockic N., Midgley J., Kenney J.T. (2010). Synthesis and characterization of CsSnI_3_ thin films. Appl. Phys. Lett..

[11] Kim Y.J., Kang D.W. (2022). The tin(ii) precursor is an active site to determine the crystal framework in CsSnI_3_ perovskite. J. Mater. Chem. A.

[12] Wang Y., Tu J., Li T., Tao C., Deng X., Li Z. (2019). Convenient preparation of CsSnI_3_ quantum dots, excellent stability, and the highest performance of lead-free inorganic perovskite solar cells so far. J. Mater. Chem. A.

[13] Murshed R., Bansal S. (2022). Additive-assisted optimization in morphology and optoelectronic properties of inorganic mixed Sn-Pb halide perovskites. Materials.

[14] Nairui X., Yehua T., Yali Q., Duoduo L., Ke-Fan W. (2020). One-step solution synthesis and stability study of inorganic perovskite semiconductor Cs_2_SnI_6_. Sol. Energy.

[15] Jiang Y., Zhang H.L., Qiu X.F., Cao B.Q. (2017). The air and thermal stabilities of lead-free perovskite variant Cs_2_SnI_6_ powder. Mater. Lett..

[16] Lee B., Krenselewski A., Baik S.I., Seidman D.N., Chang R.P.H. (2017). Solution processing of air-stable molecular semiconducting iodosalts, Cs_2_SnI_6−x_Br_x_, for potential solar cell applications. Sustain. Energ. Fuels.

[17] Saparov B., Sun J.P., Meng W.W., Xiao Z.W., Duan H.S., Gunawan O., Shin D., Hill I.G., Yan Y.F., Mitzi D.B. (2016). Thin-film deposition and characterization of a Sn-deficient perovskite derivative Cs_2_SnI_6_. Chem. Mater..

[18] Tang X., Weng S., Hao W., Yang F. (2023). Aqueous synthesis of ultrastable dual-color-emitting lead-free double-perovskite Cs_2_SnI_6_ with a wide emission span enabled by the size effect. ACS Sustain. Chem. Eng..

[19] Saski M., Prochowicz D., Marynowski W., Lewiński J. (2019). Mechanosynthesis, optical, and morphological properties of MA, FA, Cs-SnX3 (X = I, Br) and phase-pure mixed-halide MASnIxBr3–x perovskites. Eur. J. Inorg. Chem..

[20] Bharti P.C., Jha P.K., Jha P.A., Singh P. (2023). Observation of isomorphic phase transition in non-perovskite Green CsSnI_3_. Materialia.

[21] Wang A., Yan X., Zhang M., Sun S., Yang M., Shen W., Pan X., Wang P., Deng Z. (2016). Controlled synthesis of lead-free and stable perovskite derivative Cs2SnI6 nanocrystals via a facile hot-injection process. Chem. Mater..

[22] Zhou P., Chen H., Chao Y., Zhang Q., Zhang W., Lv F., Gu L., Zhao Q., Wang N., Wang J. (2021). Single-atom Pt-I3 sites on all-inorganic Cs2SnI6 perovskite for efficient photocatalytic hydrogen production. Nat. Commun..

[23] Zhu W., Xin G., Scott S.M., Xu W., Yao T., Gong B., Wang Y., Li M., Lian J. (2019). Deciphering the degradation mechanism of the lead-free all inorganic perovskite Cs2SnI6. npj Mater. Degrad..

[24] Misra R.K., Ciammaruchi L., Aharon S., Mogilyansky D., Etgar L., Visoly-Fisher I., Katz E.A. (2016). Effect of halide composition on the photochemical stability of perovskite photovoltaic materials. ChemSusChem.

[25] Chung I., Song J.-H., Im J., Androulakis J., Malliakas C.D., Li H., Freeman A.J., Kenney J.T., Kanatzidis M.G. (2012). CsSnI_3_: Semiconductor or metal? high electrical conductivity and strong near-infrared photoluminescence from a single material. high hole mobility and phase-transitions. J. Am. Chem. Soc..

[26] Wang J., Ullah S., Yang P., Liu L., Yang S., Xia T., Guo H., Chen Y. (2021). A feasible process for lead-free Cs_2_SnI_6_ films using vapor-assisted deposition method with Sn and I_2_ powders as reactants. J. Phys. D Appl. Phys..

[27] Mahon N.S., Korolik O.V., Khenkin M.V., Arnaoutakis G.E., Galagan Y., Soriūtė V., Litvinas D., Ščajev P., Katz E.A., Mazanik A.V. (2020). Photoluminescence kinetics for monitoring photoinduced processes in perovskite solar cells. Sol. Energy.

[28] Serpetzoglou E., Konidakis I., Maksudov T., Panagiotopoulos A., Kymakis E., Stratakis E. (2019). In situ monitoring of the charge carrier dynamics of CH_3_NH_3_PbI_3_ perovskite crystallization process. J. Mater. Chem. C.

[29] Leroux M., Grandjean N., Beaumont B., Nataf G., Semond F., Massies J., Gibart P. (1999). Temperature quenching of photoluminescence intensities in undoped and doped GaN. J. Appl. Phys..

[30] Yang F. (2021). Size effect on the bandgap change of quantum dots: Thermomechanical deformation. Phys. Lett. A.

[31] Tang X., Zhang Y., Kothalawala N.L., Wen X., Kim D.Y., Yang F. (2022). MAPbBr_3_ nanocrystals from aqueous solution for poly (methyl methacrylate)-MAPbBr3 nanocrystal films with compression-resistant photoluminescence. Nanotechnology.

[32] Khenkin M.V., Amasev D.V., Kozyukhin S.A., Sadovnikov A.V., Katz E.A., Kazanskii A.G. (2017). Temperature and spectral dependence of CH_3_NH_3_PbI_3_ films photoconductivity. Appl. Phys. Lett..

