# Supplementary material for: Dual Light Emission of CsSnI3-Based Powders Synthesized via a Mechanochemical Process
Source: Materials (Basel). 2024 Jul 19;17(14):3577. doi: 10.3390/ma17143577 (PMC11279061; doi:10.3390/ma17143577)
Supplement: Supplementary file 1 [file materials-17-03577-s001.zip › materials-3081442-supplementary.pdf]

Article

Dual Light Emission of $CsSnI_3$-Based Powders Synthesized via a Mechanochemical Process

Xuan Huang [1,2], Xiaobing Tang [1,3,4], Xiyu Wen [5], Yuebin Charles Lu [1,2,*] and Fuqian Yang [1,3,*]

1 Laboratory of Functional Materials, University of Kentucky, Lexington, KY 40506, USA; xuan.huang@uky.edu (X.H.); xbtang@usst.edu.cn (X.T.)
2 Department of Mechanical and Aerospace Engineering, University of Kentucky, Lexington, KY 40506, USA
3 Department of Chemical and Materials Engineering, University of Kentucky, Lexington, KY 40506, USA
4 School of Mechanical Engineering, University of Shanghai for Science and Technology, Shanghai 200093, China
5 Center for Aluminium Technology, University of Kentucky, Lexington, KY 40506, USA; xwen2@uky.edu
* Correspondence: ycharles.lu@uky.edu (Y.C.L.); fuqian.yang@uky.edu (F.Y.)

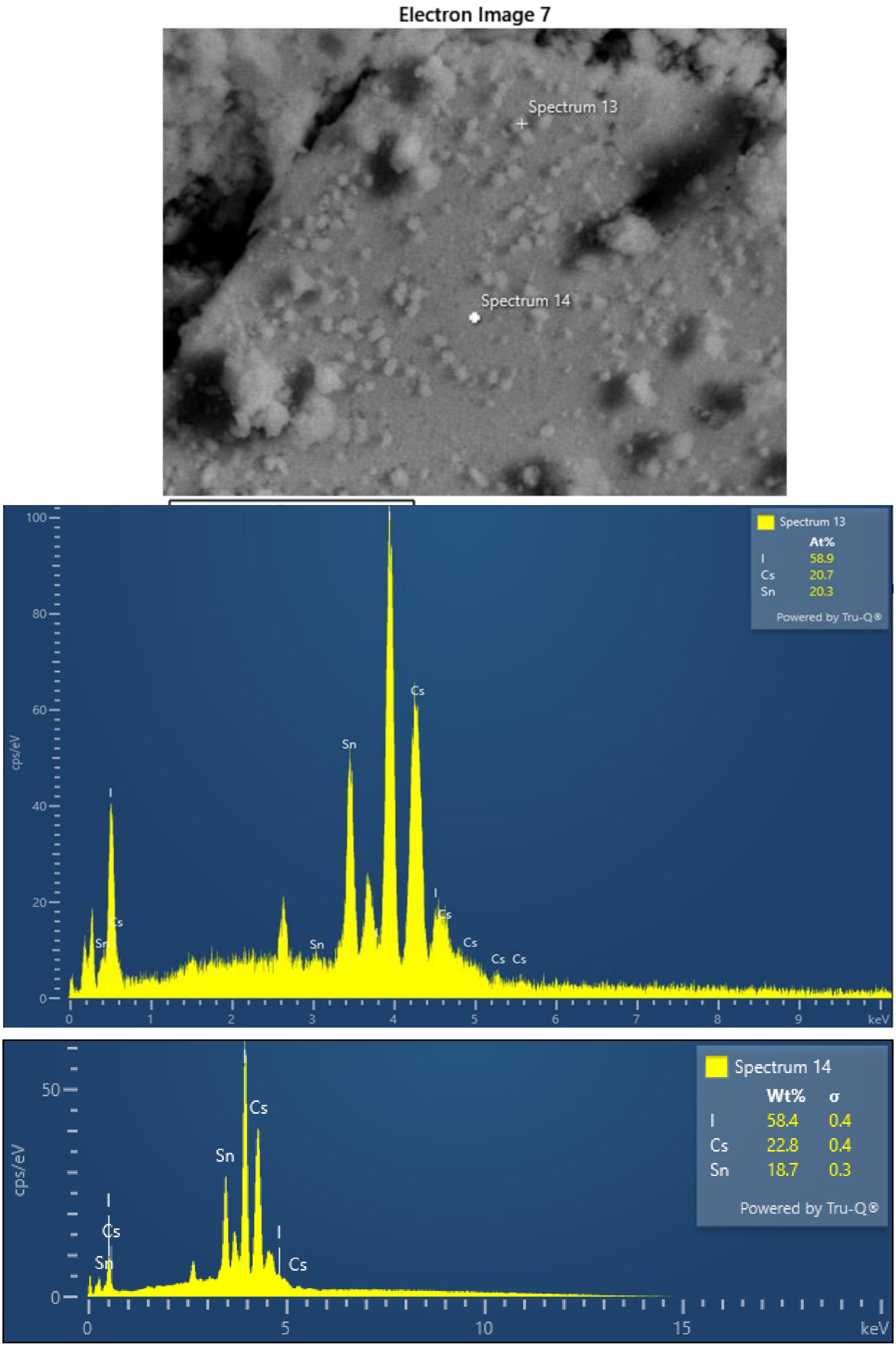

Figure S1. EDS spectrum of $CsSnI_3$ plate. (In Spectrum 14, atomic ratio of Cs: Sn: I= 21.73%: 19.96%: 58.31%).

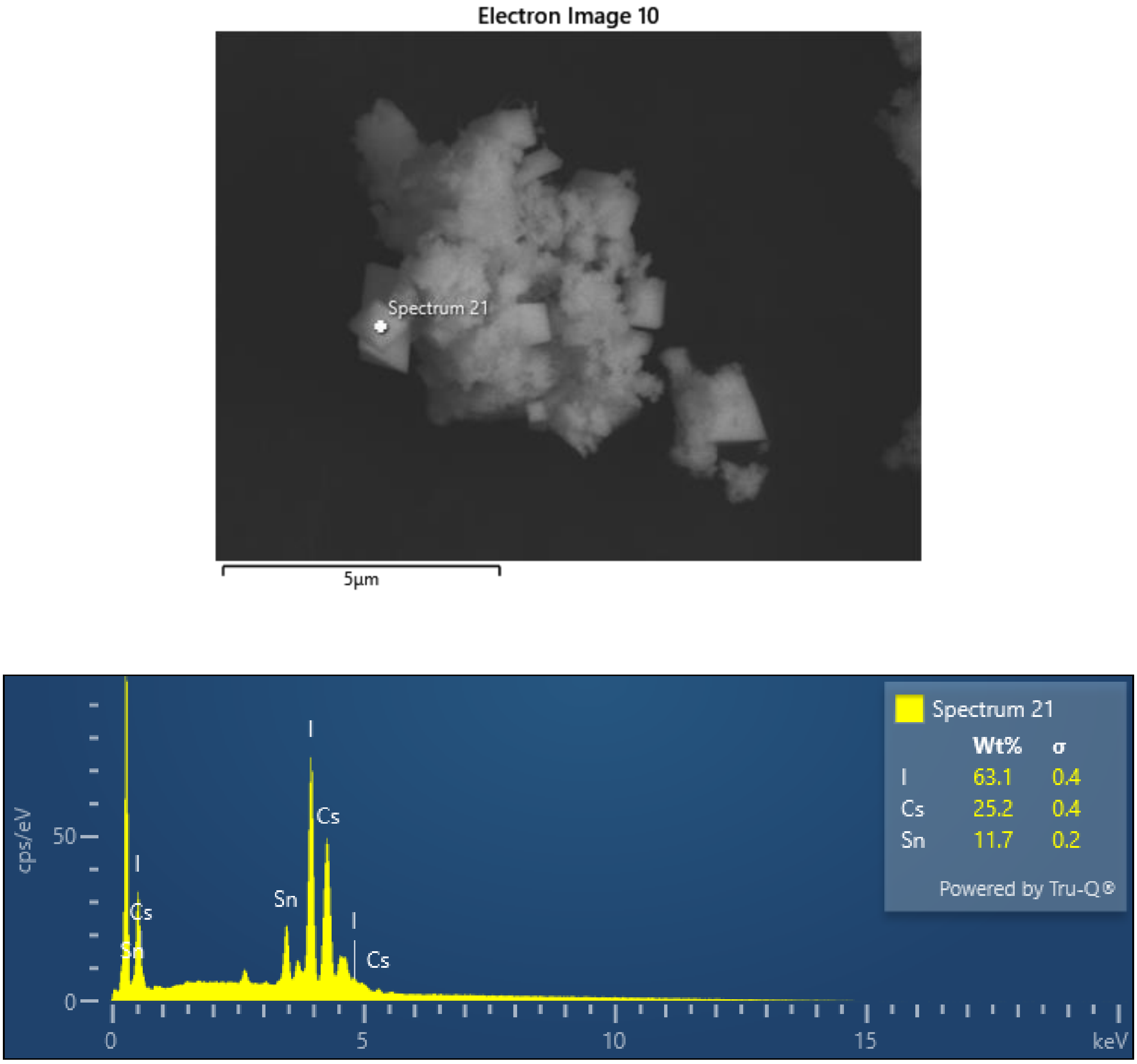

Figure S2. EDS spectrum of Cs_2SnI_6 plate. (In Spectrum 21, atomic ratio of Cs: Sn: I= 24.1%: 12.6%: 63.3%).

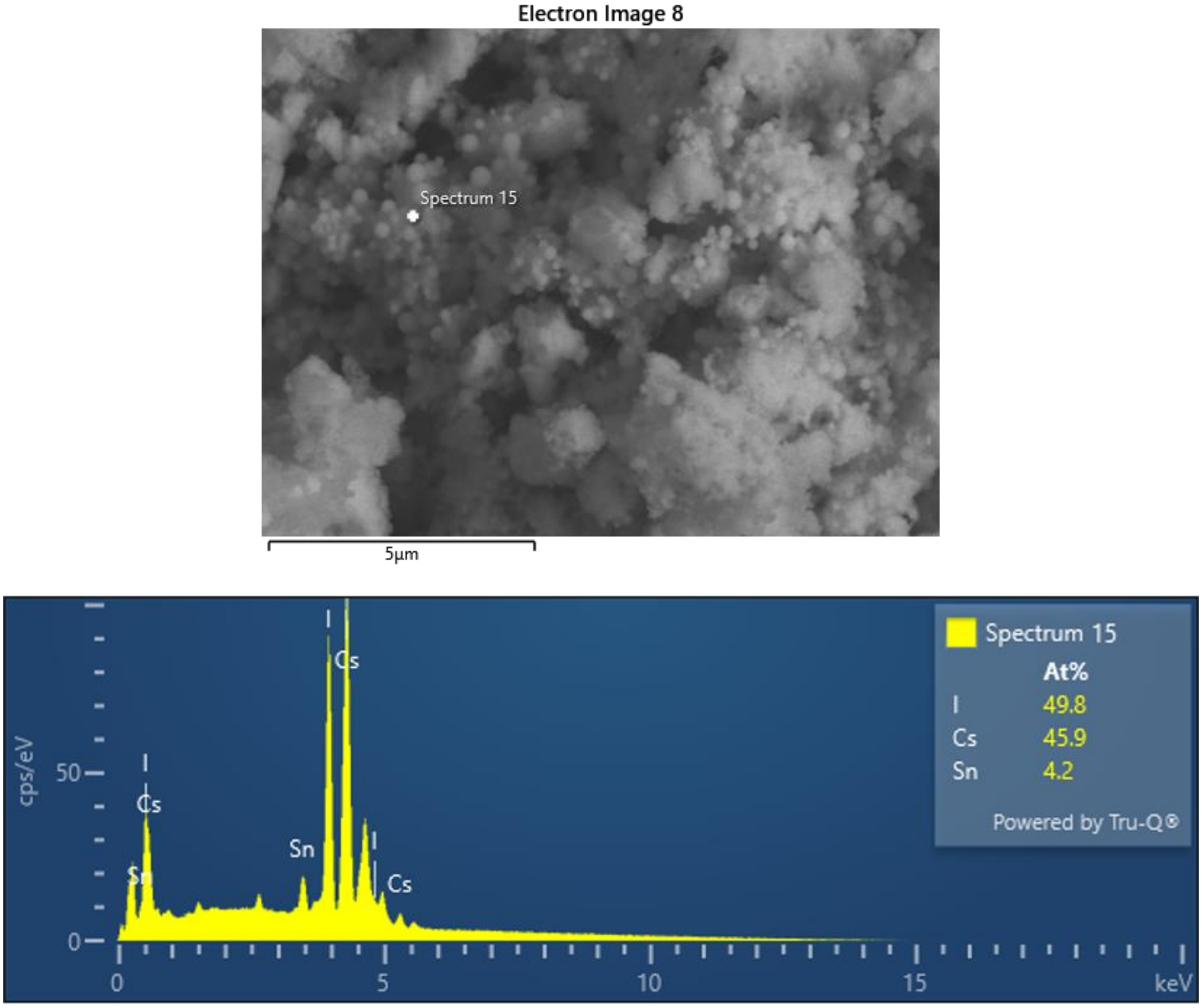

Figure S3. EDS spectrum of CsI crystals. (In Spectrum 15, atomic ratio of Cs: I= 49.80 %: 45.90 %).

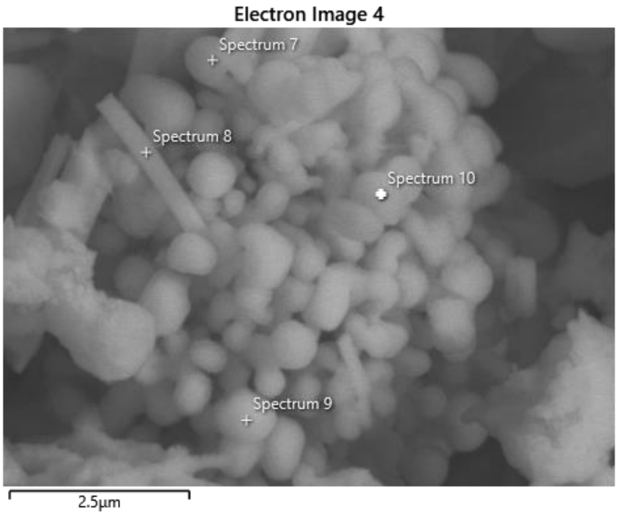

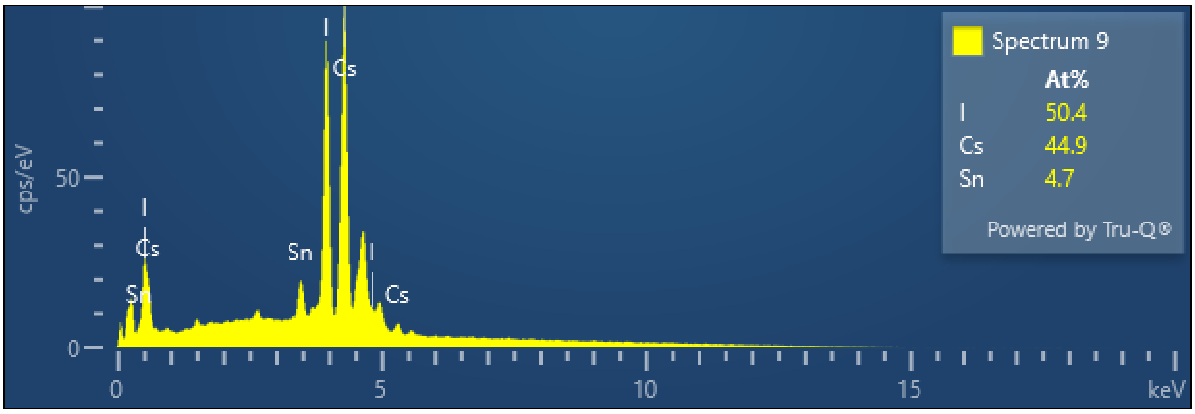

Figure S4. EDS spectrum of CsI crystals. (In Spectrum 9, atomic ratio of Cs: I= 50.40 %: 44.90 %).

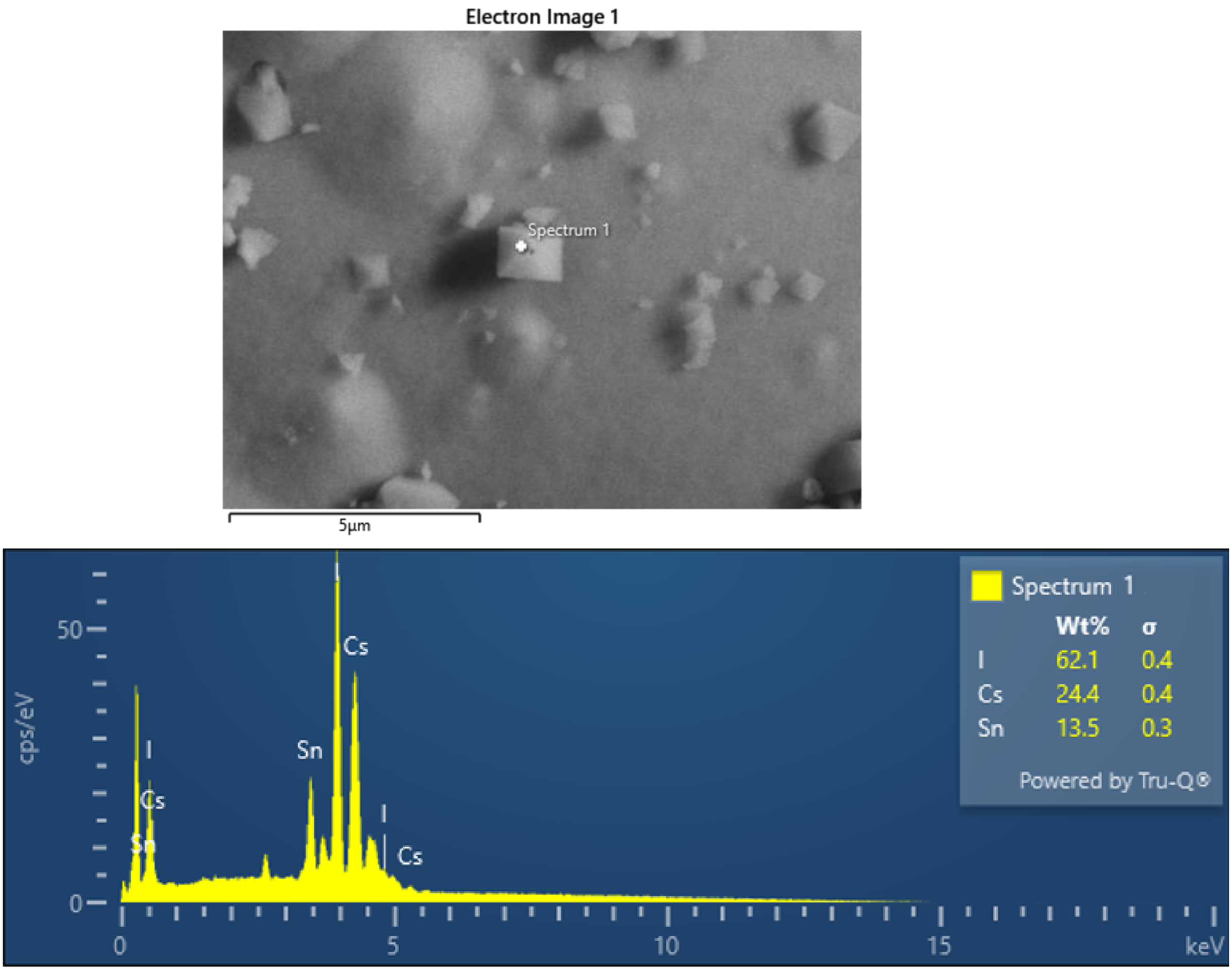

Figure S5. EDS spectrum of CsI crystals. (In Spectrum 1, atomic ratio of Cs:Sn: I= 23.34 %: 14.46 %: 62.20 %).

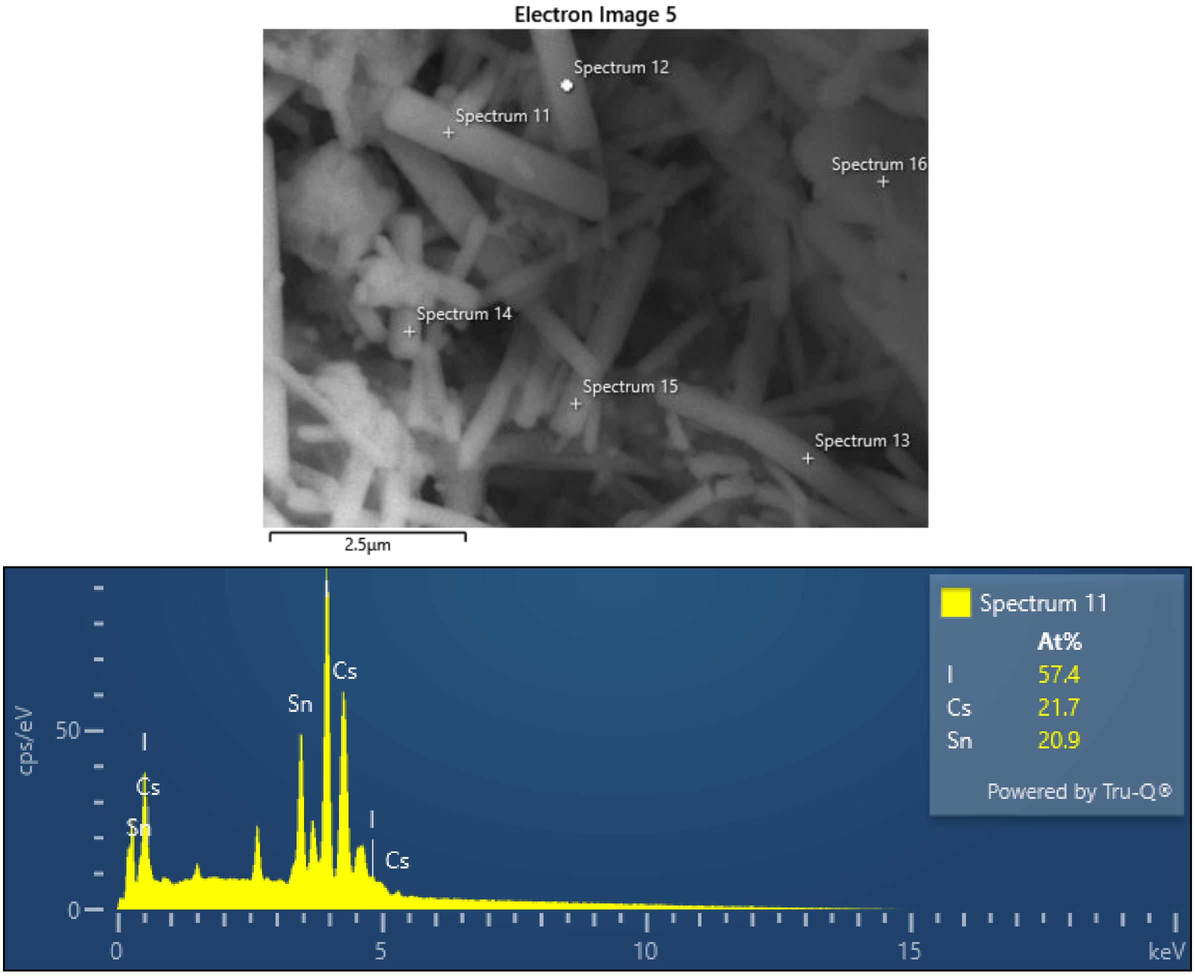

Figure S6. EDS spectrum of CsI crystals. (In Spectrum 11, atomic ratio of Cs: Sn: I= 21.7 %: 20.9 %: 57.4 %).

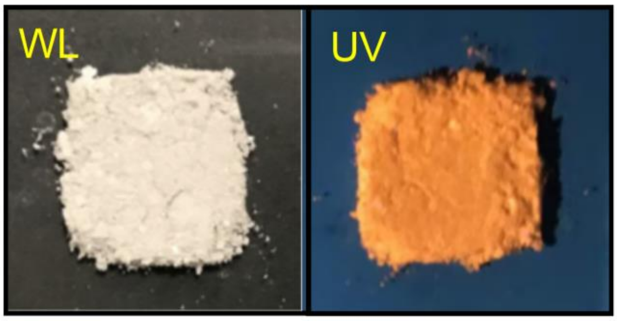

Figure S7. Optical image of SnI_2-doped CsI under white light and UV lamp (365nm). 14.9 mg (0.04 mmol) SnI_2 were stirred at 100 rpm in 3 mL DI water at 40℃ for 1 hour to produce SnI_2 suspension. 104 mg (0.4 mmol) CsI were weighed and mixed with 10 µL SnI_2 suspension in mortar (1.34×10^{-4} mmol SnI_2). Grind the mixture product for 10 minutes at a temperature of 18.4 ℃ and a humidity of 22%.

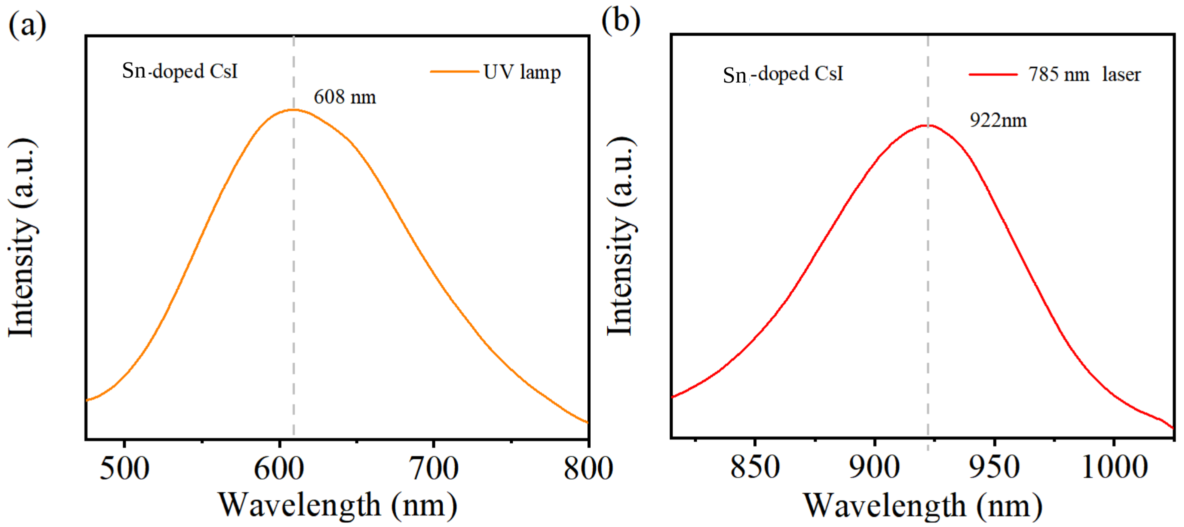

Figure S8. Photoluminescence of SnI_2-doped CsI by mechanical synthesis excited under: (a) UV lamp (365 nm); (b) 785 nm laser at 0.75v.

As shown in Figure S2, the ground product powder appears white under white light and displays 608 nm emission under UV light (365 nm). In addition, the doped CsI exhibits 922 nm infrared emission when exposed to a laser of 785 nm at 0.75 V.

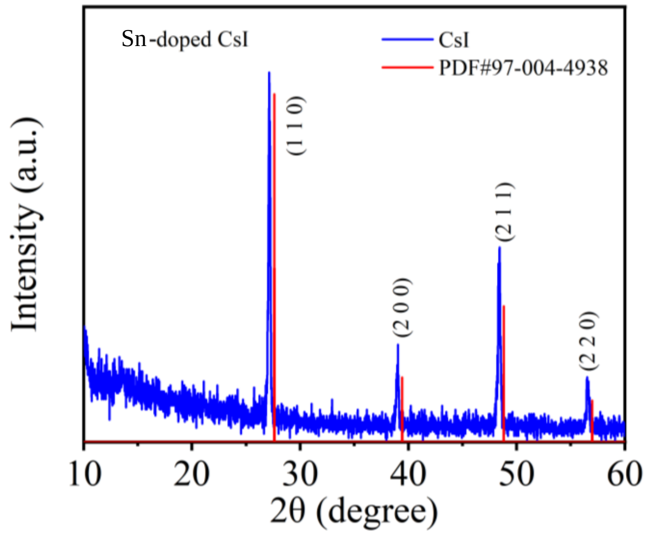

Figure S9. XRD pattern of Sn-doped CsI by mechanical synthesis.

According to Fig. S3, the Sn-doped CsI exhibits a 0.5° shift relative to the standard peaks of pure CsI, while no Cs_2SnI_6 peaks can be observed, indicating that the orange emission originates solely from Sn-doped CsI.

Table S1. Lattice constants of freshly prepared and stored $CsSnI_3$-based powders.

λ=1.5406 (Å)	h	k	l	2θ(°)	sinθ	$s=h^2+k^2+l^2$	$a=(\lambda^2/4(\sin\theta^2/s))^{0.5}$ (Å)
Fresh $CsSnI_3$	2	2	1	27.12	0.84	9	2.76
Fresh Cs_2SnI_6	2	2	2	26.32	0.56	12	4.77
Stored $CsSnI_3$	2	2	1	26.72	0.71	9	3.24
Stored Cs_2SnI_6	2	2	2	26.02	0.43	12	6.22
